# Maximizing Photoluminescence Extraction in Silicon Photonic Crystal Slabs

**DOI:** 10.1038/srep25135

**Published:** 2016-04-26

**Authors:** Ali Mahdavi, George Sarau, Jolly Xavier, Taofiq K. Paraïso, Silke Christiansen, Frank Vollmer

**Affiliations:** 1 Max Planck Institute for the Science of Light, Günther-Scharowsky-Straße 1, 91058 Erlangen, Germany; 2 Helmholtz-Zentrum Berlin für Materialien und Energie, Institute Nanoarchitectures for Energy Conversion, Hahn-Meitner-Platz 1, 14109 Berlin, Germany

## Abstract

Photonic crystal modes can be tailored for increasing light matter interactions and light extraction efficiencies. These PhC properties have been explored for improving the device performance of LEDs, solar cells and precision biosensors. Tuning the extended band structure of 2D PhC provides a means for increasing light extraction throughout a planar device. This requires careful design and fabrication of PhC with a desirable mode structure overlapping with the spectral region of emission. We show a method for predicting and maximizing light extraction from 2D photonic crystal slabs, exemplified by maximizing silicon photoluminescence (PL). Systematically varying the lattice constant and filling factor, we predict the increases in PL intensity from band structure calculations and confirm predictions in micro-PL experiments. With the near optimal design parameters of PhC, we demonstrate more than 500-fold increase in PL intensity, measured near band edge of silicon at room temperature, an enhancement by an order of magnitude more than what has been reported.

Periodic structuring of optical materials such as photonic crystal (PhC) slabs, can impart geometric control over optical modes^1^,^2^. The resulting photonic crystals modify drastically the distribution of electromagnetic modes participating to the radiation field, and can therefore be tailored to either inhibit or enhance light-matter interactions as well as light extraction efficiencies^3^,^4^,^5^. Recently, these PhC properties have been explored to improve the device performance of light emitting devices (LED’s), solar cells, precision biosensors, and devices that are compatible with microelectronic fabrication technology^6^,^7^,^8^,^9^. One particular area of intense research in the field of silicon photonics is the excitation and extraction of photoluminescence (PL)^10^. Silicon exhibits an indirect electronic band edge at ~1.12 eV (equivalent to ~1100 nm in terms of wavelength) with correspondingly weak extracted PL emission. Nano-cavities in two dimensional (2D) silicon PhCs can thereby significantly increase the measured PL intensity^11^,^12^,^13^,^14^; however, such miniscule and narrowband sources can be difficult to multiplex. Furthermore, efficient light emission results from the excitation of only a small, nanoscale area of the device, and fabricating high quality nano-cavities remains a formidable challenge^15^ Instead, tuning the extended band-structure of 2D PhCs provides another means for increasing PL intensity throughout a planar device. This requires careful design and fabrication of PhCs, with a desirable mode structure overlaps with the spectral region of PL emission.

Here we show a method for predicting and maximizing PL light extraction from defect-free 2D silicon PhC slabs patterned with triangular lattices of air holes. Systematically varying lattice constant and fill factor, we predict the rise in PL intensity from band structure calculations and confirm predictions in micro-PL experiments in a back-scattering configuration. With near optimal design parameters for the PhCs, we demonstrate a greater than 500-fold increase in PL intensity measured near the band edge of silicon at room temperature, an enhancement by an order of magnitude in respect to what has been previously reported in similar PhC structures with air holes^16^,^17^ Other studies of defect-free Si PhC slabs consist of nanorods or nanobox, have reached up to 122-fold increase in PL intensity over that of an un-patterned substrate. The quantity of radiation modes at electronic band edge are thereby identified as an important factor contributing to the enhancement of light extraction in indirect bandgap semiconductors^16^,^17^,^18^,^19^ as well as direct bandgap ones^20^,^21^.

## Photoluminescence enhancement analysis

**Figure 1:**
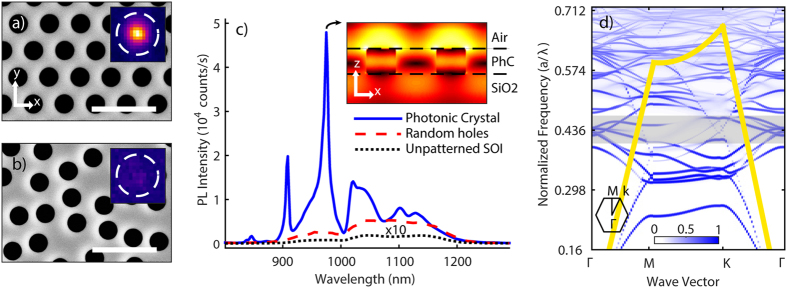
SEM micrographs of structured SOI wafers respectively of (a) PhC with *a* = 480 nm and *r/a* = 0.29 and (b) random air holes. Inset: IR camera images of PL light emission (for all camera images please refer to supplementary information S2). The scale bar is 1 μm and diameter of the dashed circles are 10 μm. (**c**) Comparison of PL emission spectra of PhC corresponding to the PhC SEM micrograph (blue), random air holes (red) and unpatterned SOI (black) multiplied by 10 for better visibility. Inset: Simulated cross-sectional (x–z) electric field intensity at 975 nm. (**d**) Simulated band structure corresponding to the PhC SEM micrograph. The gray band corresponds to wavelength range of 1100 ± 50 nm. The yellow line identifies the light line, the hexagon depicts the first Brillouin zone.

In Fig. 1a the scanning electron microscope (SEM) micrograph of an optimized PhC with geometrical parameters a = 480 nm and r/a = 0.29 is given, where *a* is lattice constant and r is hole radius, fabricated on silicon on insulator (SOI) wafers with Si slab thickness of 220 nm on a 3 μm buffered oxide layer (silica). In Fig. 1b the SEM image of nano-structured SOI with random holes fabricated for comparison is shown. PL intensity measurements are performed for both of these structures and the results are plotted in Fig. 1c. Note that a PL intensity enhancement of ~530 with respect to an unstructured SOI wafer (~760 if normalized by the area of active material) was recorded at the peak wavelength of 975 nm. In order to identify the origin of this enhancement, the PhC band structure is calculated by means of 3D Finite Difference Time Domain (FDTD, Lumerical) simulation displayed in Fig. 1d, scaled by normalized frequency *a*/*λ,* where *λ* is wavelength. In band structure, center of the radiation cone coincides with the Γ-point, the high symmetry point in the Brillouin zone where k_x_ = k_y_ = 0. At this point radiation modes couple to the objective of the micro-PL setup (schematic of the setup is in supplementary information S1), regardless of its numerical aperture (NA). The number of radiation modes around the Γ-point varies with the lattice constant and fill factor. Modes located near the Γ-point of the reciprocal lattice exhibit low group velocity and efficient coupling into the exterior, properties that leads us to predict extraction enhancement via the proportion of these modes in the band structure. In the following section, we will detail the mode density estimation of our approach and how systematic variation of design parameters can experimentally achieve optimal PL intensity enhancements.

## Structure optimization

**Figure 2:**
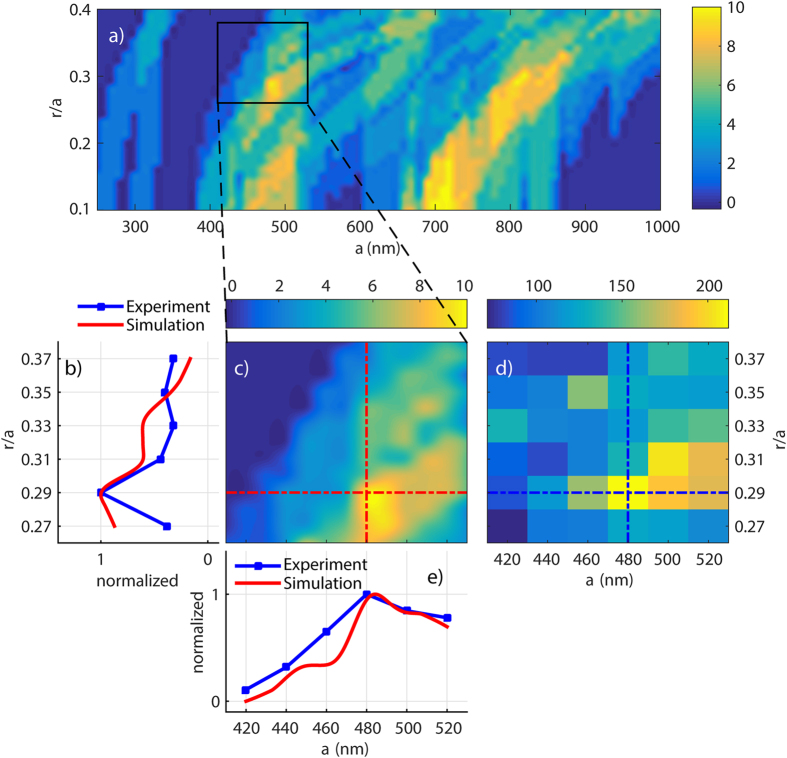
(**a**) Simulation of density of radiation modes at Γ-point of the Brillouin zone within a wavelength range of 1100 ± 50 nm, and (**c**) Zoomed-in view, corresponding to the investigated PhC structures. (**d**) Measured full spectral PL intensity enhancements (equal to the ratios of the relevant integrated PL spectra to that of the unstructured SOI wafer). (**b,e**) Normalized intensities from simulation (red) and experiment (blue).

Band structure calculations are set up using the plane-wave 3D mode solver, MPB^22^. This simulation tool returns all the k-vectors associated with transverse electric (TE) and transverse magnetic (TM) modes within the simulation domain (band structure with separate TE & TM like modes is plotted in supplementary information S3). The simulation is repeated for systematically varying lattice constant for a range of 250 < *a* < 1000 nm and normalized air hole radius of 0.1 < *r/a* < 0.4. An algorithm is established to calculate the density of the radiation modes at the Γ-point and within ± 50 nm bandwidth around the band edge of silicon at ~1100 nm. The results for this mode density calculation are displayed in Fig. 2a with clearly indicated hotspots (mode density plot). PL intensity enhancements are expected for PhC of lattice constant and fill factor lying within the hotspots shown in this map. We identify a hotspot in the mode density plot of Fig. 2a, related to a certain set of lattice parameters for which PhCs are fabricated, shown in Fig. 2c. We hereby choose the fabrication domain with lattice constant of 420 < *a* < 520 and normalized air hole radius of 0.27 < *r/a* < 0.37. This range is chosen such that the fabrication constraint of very small hole diameters can be overcome, and in the meantime a reasonable density of holes in real space is maintained with respect to the probed beam focus spot and lateral penetration of PL in Si. We fabricate a total number of 36 PhC structures (see the experimental section) to probe for PL intensity enhancements in the (*r/a, a*) parameter space selected from the mode density plot. Figure 2d shows the measured, full spectral PL intensity enhancements for the fabricated PhC structures. The full spectral PL intensity enhancements are determined by integrated PL spectrum obtained from PhC structures divided by integrated spectrum obtained from unstructured SOI wafer. A maximum full spectral PL intensity enhancement of more than 200-fold is identified, for fabricated PhC structure with lattice parameters corresponding to the hotspot identified in the mode density plot, as marked by cross bars in Fig. 2c,d. In addition, even higher enhancements are observed at PL spectral peaks obtained from PhC structures, attaining ~530-fold at the peak wavelength (to see peak enhancement of all PhC structures please refer to supplementary information S4). Comparing the experimental results with numerical methods, i.e. Fig. 2b,e, we indeed measure the highest yet unprecedented level of PL intensity enhancement, near the lattice parameters where calculations predict highest density of radiation modes. As previously discussed, Fig. 1d shows a significant concentration of flat bands within the indicated gray band near the Γ-point, corresponding to wavelengths of 1100 ± 50 nm. High quantity of flat bands with correspondingly low group velocity at the electronic band edge of Si, indicates high density of states near the Γ-point, this improves the coupling between spontaneously emitted photons and the radiation modes, and gives rise to the maximized light extraction efficiency in the direction perpendicular to the slabs^16^,^17^,^18^.

**Figure 3:**
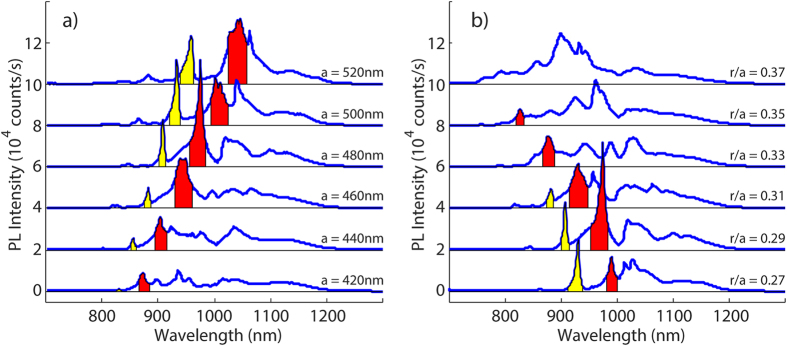
Evolution of PL spectra by varying *a* (a) and *r/a* (b). (**a**) Corresponds to the spectra of PhC structures with *r/a* = 0.29. (**b**) Corresponds to the spectra of PhC structures with *a* = 480 nm. Colored peaks are only for visual aid to observe the shift in the PL peaks.

While the PL extraction increases with the density of radiation modes near the Γ-point, it can also decrease with the density of guided modes^4^. The fact that there is no distinct peak at the silicon band edge ~1100 nm, for the particular structure shown in Fig. 1a, could also be attributed to the TE-like guided modes found within this wavelength range^23^. We also note the absorption of guided PL light in respective wavelength range. The simulated electric field distribution simulation at 975 nm radiating from a plane wave placed inside the PhC structure is shown as inset in Fig. 1c. The same plot at 1100 nm would give ~30 times less light extraction from this structure (for comparison please refer to supplementary information S5), consistent with the experimental observations. Furthermore, we discern that the particular wavelength for the highest PL peak differs from one structure to another, as it depends on lattice parameters. Approximate wavelengths for these maximal spectral PL peaks move to higher wavelengths when increasing the lattice constant, decreasing the fill factor, or both; hence, it is possible to track effective refractive index change Fig. 3.

It is well-known that by nano-structuring the surface of a light emitting device, less total internal reflection occurs and all in plane modes are more easily diffracted to the surrounding medium^24^. Here we show a comparative study between the PhC structures fabricated in SOI to that of random holes in SOI. We have fabricated random holes with the same filling fraction and the same number of holes per unit area as for the triangular PhC lattice (one example is shown in Fig. 1b). For the studied samples, the PL intensity spectrum when fill fractions were varied only led to a slight change in PL intensity, which showed inverse relation to effective refractive index of the Si material^25^. The random structures maintain the overall spectral shape of the unpatterned silicon PL, and no peaks or other spectral features are resolved. Imaging of PL light extracted from PhC structures and from random air hole structures are shown in comparison in the inset of micrographs in Fig. 1a,b, respectively. For the fabricated random air holes in SOI, we observe a maximum of 40-fold full spectral enhancement as compared to the unpatterned SOI wafer. Comparing PL spectrum of PhC structures to that of the randomized variants with the same fill fraction allows us to estimate the contribution of the PhC band structure to PL light enhancement Fig. 1c. It is to be noted that the primary factors contributing to this enhancement are the density of optical modes and extraction efficiency^26^. Considering limited numerical aperture of the collection optics, tailoring the radiation pattern in order to optimally collect the emission is also important. The most critical factor, however, for enhanced spontaneous emission output in the PhCs appears to be the extraction efficiency by Bragg scattering in to the escape cone^25^. While a random pattern might also fulfil this condition, PhC structure can introduce additional enhancement by tuning the optical modes and thus, result in orders of magnitude enhancement of relative PL extraction, as is shown in this study.

## Temperature tuning of PL spectra

**Figure 4:**
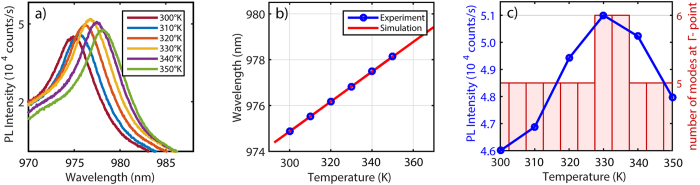
(**a**) Experimental PL spectral peak variation versus temperature. (**b**) Comparison of experimental and simulated values of the PL peak wavelength. (**c**) PL peak intensity versus calculated number of modes at the Γ-point, within the 1100 ± 20 nm wavelength range.

Since crystalline silicon’s poor quantum efficiency is due to the predominantly phonon-assisted nature of the process^27^, enhancing the interband light emission can be achieved by varying the temperature. Tuning the temperature causes thermal expansion but, most importantly, it alters the refractive index of the material as well. While temperature dependence of the refractive index of silicon^28^ and silica^29^ as well as their linear expansion are well studied, few reports exist on influence of temperature on PL emission of PhC^30^. In this section, we analyze the temperature dependence of the PhC under consideration (a = 480 nm and r/a = 0.29). The sample is placed on a temperature controlled stage and PL spectra are recorded for every 10 degree from 300 °K (room temperature) to 350 °K shown in Fig. 4a. Our focus here is on the dominant peak of this spectrum which at room temperature is at 975 nm. By increasing temperature this peak shows shift in peak wavelength as well as peak intensity variation. To simulate band structure at different temperatures, we must also know the thermal expansion coefficient (α) and temperature dependence of the refractive index (β) of the SOI wafer: *α*(*T*) = (1/*h*)(*dh*/*dT*) and *β*(*T*) = (1/*n*)(*dn*/*dT*)^28^, where *h* is length i.e. lattice constant, *n* is refractive index and *T* is temperature (for detailed graphs of *α*(*T*) and *β*(*T*) please refer to supplementary information S6). By putting these variables in to account, a linear increase in the wavelength of the band (correlated with the peak at 975 nm) is observed. Such a trend is consistent with experiments, whose data are shown in Fig. 4b.

As mentioned earlier, overall intensity of the extracted PL is related to mode concentration at the Γ-point of the Brillouin zone in a wavelength range that coincides with the electronic band gap of silicon. The quantity of radiation modes at Γ-point, within a ± 20 nm bandwidth around 1100 nm and at 330 °K as well as 335 °K, corresponds to 6 and for remaining temperatures, this number falls to 5; therefore, a higher PL extraction around 330 °K and 335 °K is expected as shown in Fig. 4c. A narrower bandwidth is chosen here due to the very subtle fluctuations in bands within this temperature range. We find that although the change of peak wavelength is dominated by the temperature dependence of the refractive index, the thermal expansion cannot be ignored. Regardless the mode density method used in this study is applicable to both subtle and drastic PL intensity variations.

## Conclusions

In summary, we demonstrate a robust all-silicon PL device that uses a tailored photonic band structure to enhance PL in a near-infrared wavelength range from 800 nm to 1100 nm. More than a 500-fold increase in extracted PL light in the systematically designed 220 nm thick SOI structures is reported. Robustness of the structure is ensured by the fabrication of a photonic crystal slab without the need of under-etching, thus maintaining a high degree of mechanical stability. Experimental measurement technique is noninvasive as samples are probed from free-space in a backscattering configuration. Samples display very stable PL peaks, and because the design of low dispersive band at the Γ-point circumvents the need of a cavity, the entire surface of PhC structure can be used for enhancing light interactions and extractions. Such spatially unconstrained enhancement is crucial for designing efficient light sources, solar cells and large area biosensors.

## Methods

Samples were fabricated on SOI wafers purchased from SOITEC. The top-most 220 nm thick p-doped silicon <100> device layer is separated from the thick silicon substrate by a 3 μm thick silicon-oxide insulating (buried-oxide) layer. Wafers are spin-coated with positive e-beam resist ZEP520A and baked at 180 °C for 3 min. The resist is exposed with the photonic crystal pattern in a RAITH 100 keV e-beam lithography system. After developing the resist, the pattern is subsequently transferred to the Si using an ICP-RIE quasi-Bosch fluorine etch process. The excess e-beam resist is removed by cascaded immersion into trichloroethylene and solvents and the wafers are finally cleaned in a Piranha solution (3:1 H2SO4:H2O2) at 120 °C^31^.

The investigated structures are defectless 2D triangular lattice of air hole PhCs etched into the SOI wafer. In total, 36 samples with different lattice parameters are fabricated with lattice constant varying from 420 to 520 nm with increments of 20 nm, and normalized air radius varying from 0.27 to 0.37 with increments of 0.02. The buried silica layer is not removed to allow better heat dissipation as compared to the air-bridged samples. The reflection of the silica layer is very low at the 457 nm excitation wavelength and the presence of the silicon layer on top reduces this significantly therefore the reflection from silica layer is negligible in our studies.

A microphotoluminescence setup is used for probing the PL intensity of the crystalline silicon and silicon PhC structures. The structures are excited with a continues wave solid state laser at 457 nm with 0.85 mW intensity power, focused through a 0.75 NA 50× microscope objective into ~1.5 μm diameter excitation spots. The two other tested wavelengths were 532 nm and 660 nm yet, due to general invariance in the observed PL signals, only the results for 457 nm are shown. The emitted PL intensity is spectrally resolved with a 50 Å resolution with two separate detectors embedded in the setup, a silicon CCD photodetector, spanning from the visible up to 1000 nm and an InGaAs nitrogen-cooled photodetector, for the near infrared (NIR) regime covering wavelengths above 1000 nm. The spectrometer is also equipped with an InGaAs IR camera. Thermal coefficients, *α* and *β*, used for Si are 2.5247e-6 (1/*K*) and 5.542e-5 (1/*K*) respectively and the same parameters for silica are 2.5247e-6 (1/*K*) and 8.7e-6 (1/*K*), respectively as measured at 293 °*K*.

## Additional Information

**How to cite this article**: Mahdavi, A. *et al*. Maximizing Photoluminescence Extraction in Silicon Photonic Crystal Slabs. *Sci. Rep.* **6**, 25135; doi: 10.1038/srep25135 (2016).

## Supplementary Material

Supplementary Information
