# Supplementary material for: Maximizing Photoluminescence Extraction in Silicon Photonic Crystal Slabs
Source: Sci Rep. 2016 Apr 26;6:25135. doi: 10.1038/srep25135 (PMC4844958; doi:10.1038/srep25135)
Supplement: Supplementary Information [file srep25135-s1.pdf]

Maximizing Photoluminescence Extraction in Silicon Photonic Crystal Slabs

Ali Mahdavi[1,*], George Sarau[1,2], Jolly Xavier[1], Taofiq Paraiso[1], Silke Christiansen[1,2], Frank Vollmer[1,#]

[1] Max Planck Institute for the Science of Light, Günther-Scharowsky-Straße 1, 91058 Erlangen, Germany.
[2] Helmholtz-Zentrum Berlin für Materialien und Energie, Institute Nanoarchitectures for Energy Conversion, Hahn-Meitner-Platz 1, 14109 Berlin/Germany
[*]email: ali.mahdavi@mpl.mpg.de
[#]corresponding author: frank.vollmer@mpl.mpg.de

Supplementary information S1: Schematic of the measurement setup: The pump CW laser with wavelength of 457nm is focused on top of the PhC structures with an objective (50x, NA 0.75). Photoluminescent emission spectra are collected by the same objective in the back-scattering configuration, passed through a VIS-NIR beam-splitter and analyzed by a NIR spectrometer equipped with an InGaAs detector. Instead of using the spectrometer, the collected light is also passed through an edge filter (750 nm cut on wavelength) and focused on an InGaAs infrared camera.

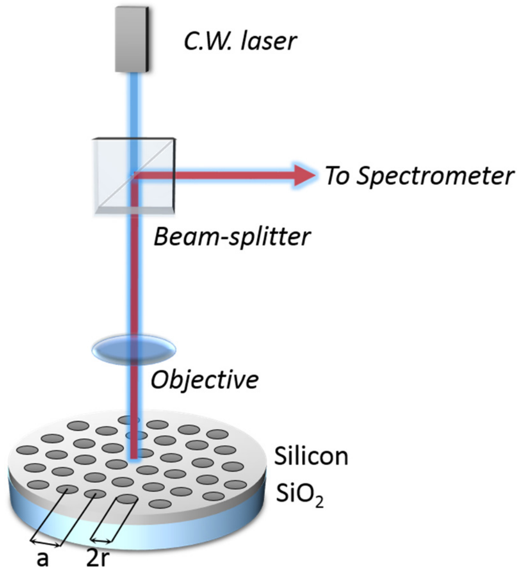

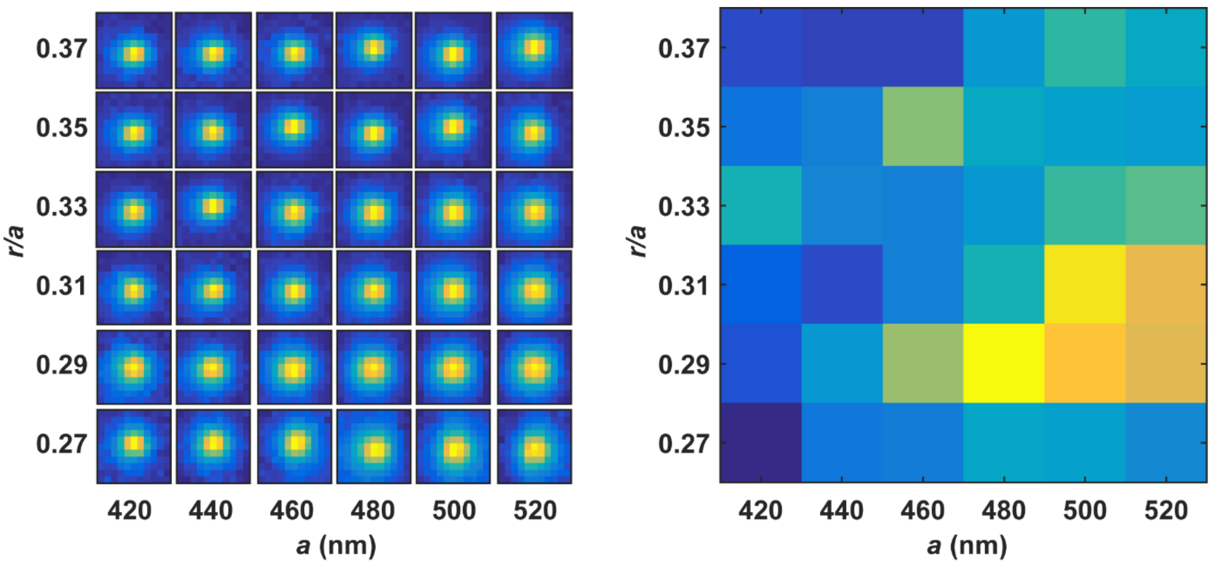

Supplementary information S2: Imaging of the PL light extraction enhancement with the infrared camera provides visual aid for further confirmation of light enhancement and its excitation profile in the PhC structure.

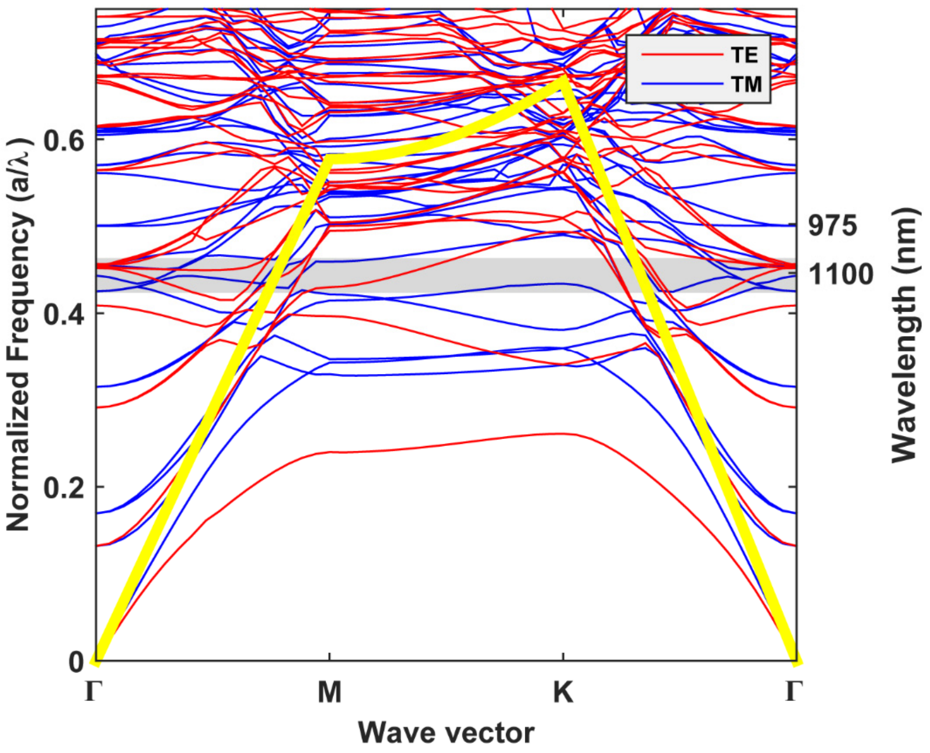

Supplementary information S3: Simulated bandstructure corresponding to the PhC structure with a = 480 nm and r/a = 0.29 using the plane-wave 3D mode solver, MPB. This simulation tool returns all the k-vectors associated with transverse electric (TE) and transverse magnetic (TM) modes.

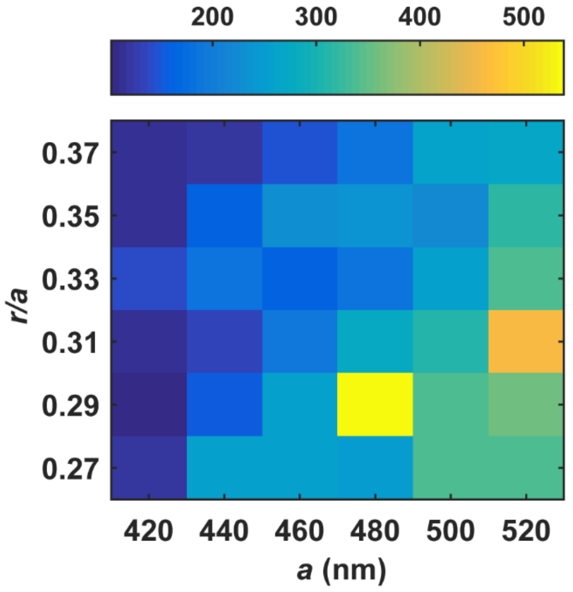

Supplementary information S4: Spectral PL peak intensity enhancements, equal to the ratios of the relevant PL peak intensity for each PhC structure to that of the unstructured SOI wafer.

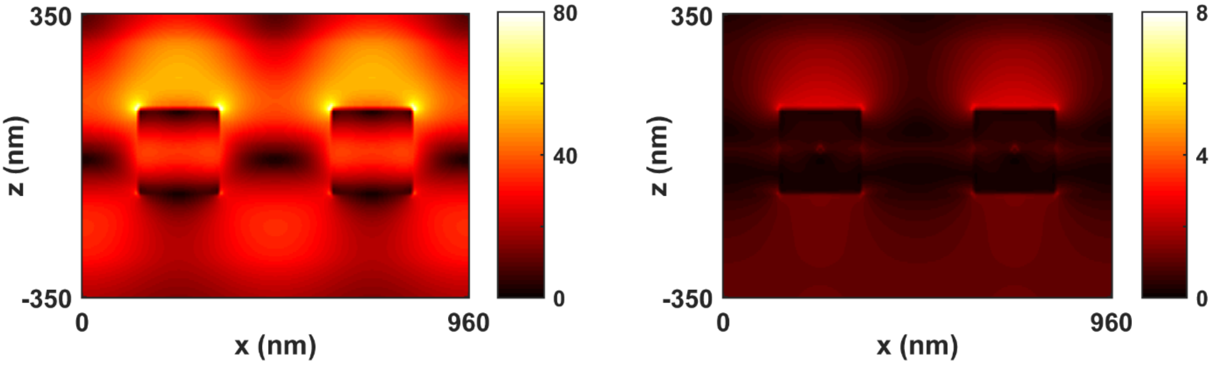

Supplementary information S5: Simulated cross-sectional (x-z) electric field intensity at 975 nm (left) and at 1100 nm (right). Note that the maximum field intensity is 82.7 on the left whereas this number corresponds to 2.8 on the right.

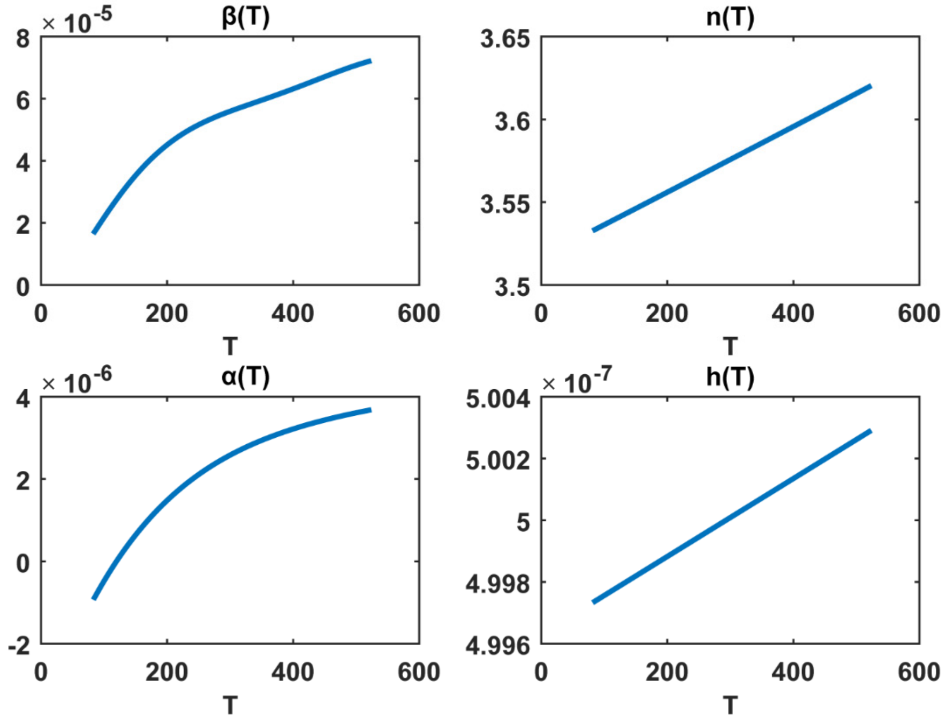

Supplementary information S6: Extracted values of $\beta(T)$ and $\alpha(T)$ of Si at 1.15 µm and the calculated n and h.
